# Laparoscopic Excision of a Symptomatic Urachal Cyst (UC) in an Adult

**DOI:** 10.7759/cureus.40846

**Published:** 2023-06-23

**Authors:** Emil J Mathew, Sandra S Kunnel, Maya Devi, Sanoop K Zachariah

**Affiliations:** 1 General and Minimal Access Surgery, Kerala Institute of Medical Sciences (KIMS HEALTH), Trivandrum, IND; 2 Surgery, Pushpagiri Institute of Medical Sciences & Research Centre, Thiruvilla, IND

**Keywords:** umbilical cyst, umbilicus, laparoscopy, urachal anomaly, adult, urachal cyst

## Abstract

Urachal cysts (UCs) are rare congenital anomalies, especially in adults. They often mimic a variety of intra-abdominal pathologies, making the diagnosis difficult. Laparotomy and excision of the cyst along with the umbilicus has been the traditional technique. A 33-year-old female presented with painful umbilical swelling. A CT scan was suggestive of a UC. We performed an umbilicus-preserving laparoscopic excision of the cyst. We describe this rare case and review literature related to the surgical treatment of UCs.

## Introduction

The true incidence of urachal anomalies as well as urachal cysts (UCs) is largely unknown. Ucs are uncommon anomalies that are generally seen in children and very rarely if ever present in adulthood [1].

Retrospective studies of small samples of pediatric autopsy records and radiological databases show that 1.03% of the general pediatric population was found to have urachal anomalies. The estimated incidence of UCs is 1 in 5000 births, with a ratio of 2:1 for males compared to females [2-3].

Urachal cysts go undetected as unless they are infected, they usually remain asymptomatic. When infected, patients can present with various non-specific symptoms such as abdominal pain, fever, umbilical discharge, and the sensation of a midline mass in the abdomen. Appendicitis, diverticulitis, urinary tract infection, pelvic inflammatory disease, and bladder carcinoma are some of the conditions that are common misdiagnoses due to the overlap in symptomatology [4].

We report a rare case of a UC in a 33-year-old female that was successfully managed by laparoscopic surgery.

## Case presentation

**Figure 1:**
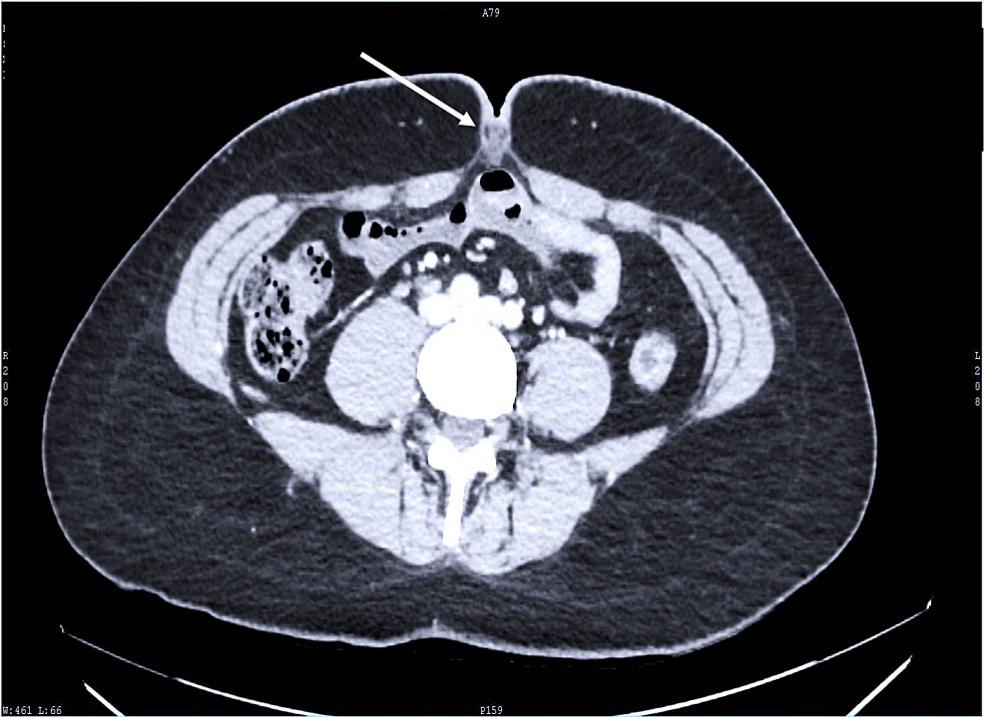
CT scan axial view showing the UC as depicted by the white arrow. UC, urachal cyst

A 33-year-old married female who was generally free of any ailments presented to us with painful umbilical swelling for a few days duration. Her body mass index (BMI) was 28.3 kg/m2. Clinical examination revealed a tender swelling about 1.5 cm in diameter within the umbilicus. Initial abdominal ultrasonography showed features suggestive of a cystic swelling in the sub-umbilical plane with no evidence of communication with the peritoneal cavity with the tentative diagnosis of a UC. An abdominal contrast enhanced computed tomography (CECT) scan elucidated a non-enhancing lesion measuring 2.3 cm x 1.9 cm with imperceptible walls further suggesting a diagnosis of UC (Figure 1).

**Figure 2:**
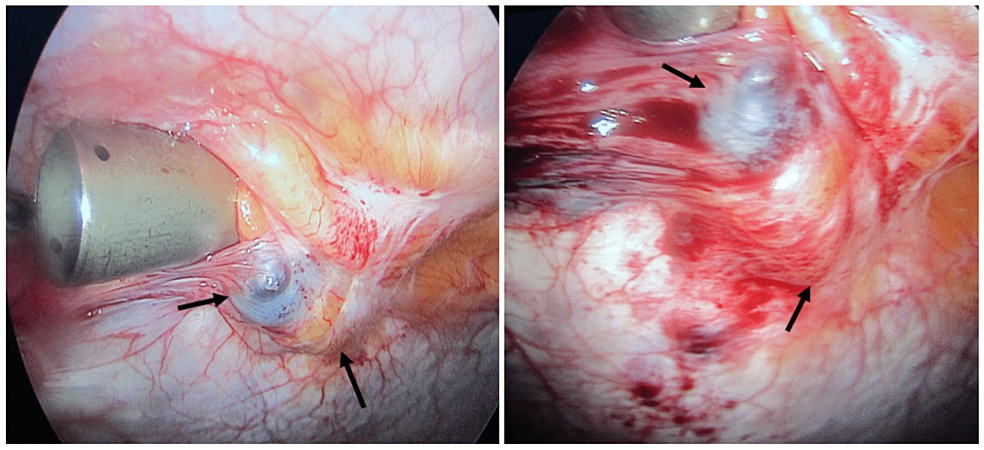
Operative photograph showing the UC as depicted by the black arrows. UC, urachal cyst

The preprocedural blood workup was unremarkable. We proceeded to perform a laparoscopic excision of the cyst under general anesthesia. The patient was placed in a supine position with the operating surgeon to the left of the patient. The entry and creation of pneumoperitoneum were made at the supra-umbilical cicatrix using an open technique Three ports were used -- a 10-mm camera port and two 5-mm working ports at the lateral border of the rectus abdominous muscle. A 30-degree laparoscope was used. An initial survey showed a brownish-gray spherical cyst about 2 cm in size below in the sub-umbilical region (Figure 2).

**Figure 3:**
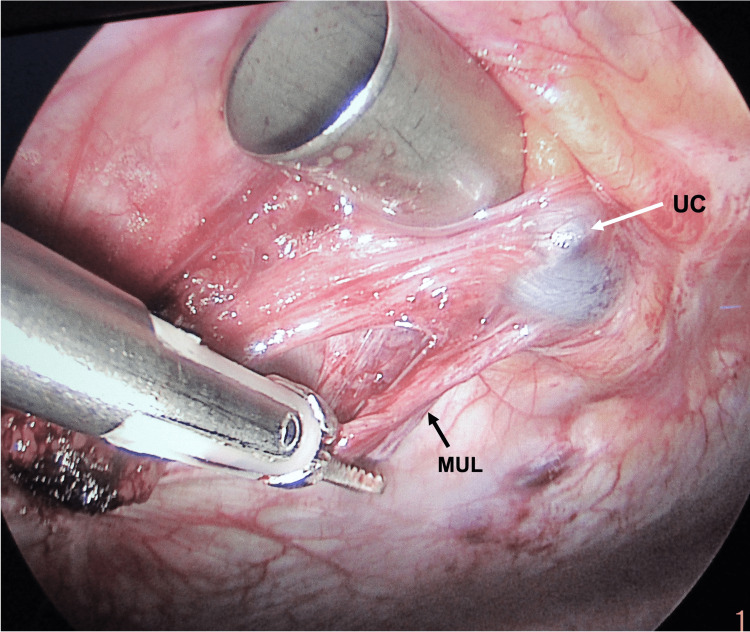
The MUL being mobilized to be excised along with the UC. The trocar is being pushed upwards to provide counter traction on the abdominal wall to facilitate dissection. MUL, median umbilical ligament; UC, urachal cyst

The rest of the abdominal viscera appeared normal. The cyst along with the fibrotic band (median umbilical ligament) extending from the cyst's inferior aspect up to the bladder's dome was excised (Figure 3).

A trocar was retained at the entry point to provide counter traction to the abdominal wall during the dissection of the cyst (Figures 2-3) while the dissection was facilitated with a monopolar hook and HARMONIC® Scalpel Shears (Ethicon Endo-Surgery, LLC, Guaynabo, Puerto Ricco 00969 USA).

No obvious bladder involvement was found. Two endo-clips were applied close to its insertion in the bladder. The specimen was retrieved in an endo-bag. The operating time was approximately 90 min with minimal blood loss. The entry defect was closed with a port closure suture. The postoperative period was uneventful, and the patient was doing well up to 6 months of follow-up. The histopathology report confirmed the diagnosis of the UC with no evidence of malignancy.

## Discussion

The urachus is a fibrous vestigial cord connecting the urinary bladder's dome to the umbilicus. It is the remnant of the fetal allantois and is postulated to serve as a channel for the excretion of fetal nitrogenous waste from the placenta through the umbilical cord. 

The fetal bladder initially lies at the level of the umbilicus. Later, during the fourth and fifth months of gestation as the bladder descends towards the pelvis the urachus gets stretched. In the course of time, the lumen gets completely obliterated to form a fibromuscular cord connecting the anterior bladder to the umbilicus. This completely obliterated urachus is now known as MUL. The MUL is a vestigial structure with no known function. Improper or incomplete obliteration of the urachus can occur and this leads to the development of various urachal anomalies as some or the whole of the channel remains patent [5].

**Figure 4:**
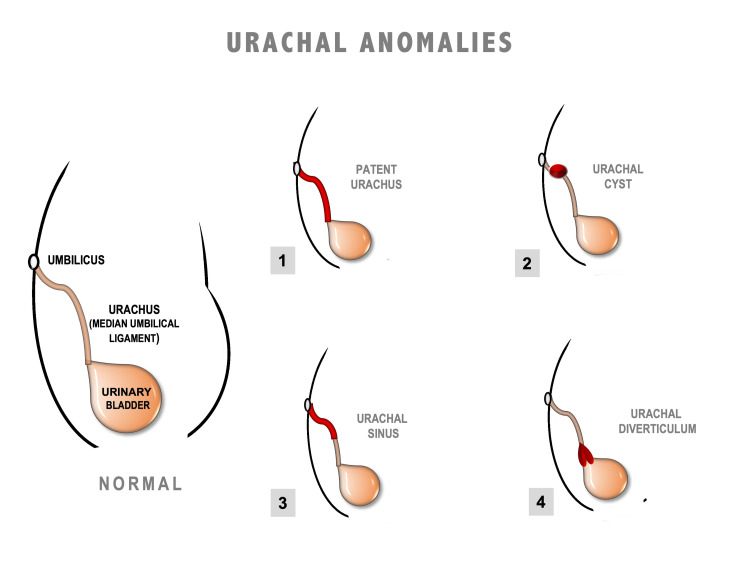
Schematic representation of the four types of urachal anomalies depending on the level of obliteration. The red portions denote the non-obliterated part of the urachus. This original illustration has been created by the authors with the Microsoft PowerPoint tool.

Four types of anomalies have been described by Bauer and Retik [6], depending on the level of obliteration and patency. This can lead to the formation of a patent urachus, cyst, sinus, or diverticulum, as depicted in the image (Figure 4).

Morphologically the urachus is between 3-10 cm long and 8-10 mm in diameter and is composed of three layers. The innermost layer is transitional epithelium in 70% and columnar epithelium in the remaining 30%. A submucosal middle layer surrounds this. The outermost layer is muscular and continuous with the detrusor muscle of the bladder. All these layers are converted to fibrous tissue once the lumen is obliterated [7-8]. Desquamation and degeneration of the urachal epithelium may result in cyst formation. The connection to the bladder may predispose to cyst infection. Rupture of the infected cysts may lead to serious conditions such as peritonitis and rarely necrotizing fasciitis [9-10].

Owing to the lack of specific symptoms, arriving at an accurate diagnosis necessitates a thorough history as well as a complete physical examination coupled with radiological evaluation. Ultrasonography and CT scans usually are the investigations of choice for UCs. A CT scan can be helpful to detect the presence of a malignancy within the cyst [11].

The definitive treatment option for UCs is surgical excision. Initial non-operative treatment for infected UCs includes aspiration and antibiotic therapy before a definitive excision, however, this may be associated with a 30% recurrence rate [12]. An open surgical excision sometimes with the sacrifice of the umbilicus used to be the traditional technique. Laparoscopic minimally invasive procedures including robotic surgery have been gaining popularity and acceptance as a safe and effective alternative to open surgery.

In our patient, we found that the laparoscopic procedure provided an excellent view of the operative field and the UC which could be dissected off the undersurface of the umbilicus thus helping to preserve it.

## Conclusions

The clinical diagnosis of UCs in adults is based on a high index of clinical suspicion. Ultrasonography and CT are often the investigations of choice. Laparoscopic excision can be considered a safe and effective option for the complete excision of UCs. 
